# A randomized controlled trial of acupuncture and receptive music therapy for sleep disorders in the elderly—ELAMUS: study protocol

**DOI:** 10.1186/s12906-024-04581-4

**Published:** 2024-08-02

**Authors:** C. Klocke, K. Rhein, H. Cramer, B. Kröger, A-J. Wetzel, J. Vagedes, H. Mauch, F. Beißner, S. Joos, J. Valentini

**Affiliations:** 1 Institute of General Practice and Interprofessional Care, University Hospital and Faculty of Medicine Tübingen, Tübingen, Germany; 2 Robert Bosch Center for Integrative Medicine and Health, Bosch Health Campus, Stuttgart, Germany; 3 ARCIM Institute, Filderklinik, Filderstadt, Germany; 4 Department of Neonatology, University Hospital and Faculty of Medicine Tübingen, Tübingen, Germany; 5 Insula Institute for Integrative Therapy Research, Hannover, Germany

**Keywords:** Sleep disorders, Acupuncture, Music therapy, Elderly people, Multimodal approach, Traditional Chinese Medicine

## Abstract

**Background:**

Globally, the demographic shift towards an aging population leads to significant challenges in healthcare systems, specifically due to an increasing incidence of multimorbidity resulting in polypharmacy among the elderly. Simultaneously, sleep disorders are a common complaint for elderly people. A treatment with pharmacological therapies often leads to side effects causing a high potential for dependency. Within this context, there is a high need to explore non-pharmacological therapeutic approaches. The purpose of this study is to evaluate the effectiveness of acupuncture and music therapy, both individually and combined as a multimodal therapy, in the treatment of sleep disorders in individuals aged 70 years and older.

**Methods:**

We conduct a confirmatory randomized controlled trial using a two-factorial study design. A total of *n* = 100 elderly people receive evidence-based standard care information for age-related sleep disorders. Beyond that, patients are randomly assigned into four groups of *n* = 25 each to receive acupuncture, receptive music therapy with a monochord, multimodal therapy with both acupuncture and music therapy, or no further therapy. The study’s primary outcome measurement is the improvement in sleep quality as assessed by the Pittsburgh Sleep Quality Index (PSQI) (global score), at the end of intervention. Additionally, depression scores (Geriatric Depression Scale), health-related quality of life (Short-Form-Health Survey-12), neurovegetative activity measured via heart rate variability, and safety data are collected as secondary outcomes. Using a mixed-methods approach, a qualitative process evaluation will be conducted to complement the quantitative data.

**Discussion:**

The study is ongoing and the last patient in is expected to be enrolled in April 2024. The results can provide valuable insights into the effectiveness of non-pharmacological interventions for sleep disorders among the elderly, contributing to a more personalized and holistic approach in geriatric healthcare.

**Trial registration:**

German Clinical Trials Register (DRKS00031886).

**Supplementary Information:**

The online version contains supplementary material available at 10.1186/s12906-024-04581-4.

## Background

For several decades, a shift in the global population structure is ongoing. An increasing life expectancy with a decline in mortality at an older age converge with a declining birth rate, which leads to an increasing proportion of older people in many countries [1]. This demographic shift comes with a multitude of challenges for our healthcare systems. One of the main problems is multimorbidity, which means the simultaneous existence or occurrence of several chronic diseases requiring treatment in one person [2–5]. The number of diseases and the proportion of people with multimorbidity increase significantly with age; by the age of 50, almost half of the population shows at least one disease. By the age of 65, most people are already suffering from multimorbidity [6]. In the Berlin age study, 88 percent of all people aged 70 years were diagnosed with more than five diseases at the same time [7–10]. Multimorbidity usually leads to the simultaneous use of several medications, which is often referred to as multimedication or polypharmacy [11–13]. According to the 2012 healthcare report, 42 percent of all patients over the age of 65 are on multiple medications, with five or more prescriptions within a quarter [14]. This leads to a frequent occurrence of drug side effects and interactions as well as misuse and reduced adherence, with the risk increasing exponentially with the amount of medication [15, 16]. Potential complications of multimedication in the elderly can include general complaints such as drowsiness, confusion, dizziness, nausea and falls with serious injuries. Also, increased risk of fatal bleeding can occur when taking anticoagulants [17, 18].

The German Society for Geriatrics (DGG), the German Society for Gerontology and Geriatrics (DGGG) and the Federal Working Group of Clinical Geriatric Institutions (BAG) define geriatric patients as patients 80 years and older or patients aged 70 years and older with a concurrent age-related multimorbidity [19]. One of the most common complaints in geriatric patients, which can aggravate existing symptoms and the incidence of polypharmacy, is sleep disturbance. The incidence and prevalence of sleep disorders increase with age, with around ten percent of men and twenty percent of women over the age of 75 suffering from them [20]. Long-term sleep disorders are associated with an increased risk of cardiovascular disease, chronic illness, depression and increased mortality [21–25]. Available drug therapies often have side effects and/or a high dependency potential (e.g. Z-drugs, benzodiazepines), especially for geriatric patients [16, 26].

Within this context, there is an urgent need for research considering more targeted non-pharmacological strategies for the medical care of ageing people. Complementary and integrative medicine (CIM) offers a wide range of methods. Some of these approaches have not yet been sufficiently studied, while others are already well investigated and, in some cases, recommended in guidelines but have not yet been sufficiently implemented in everyday care for geriatric patients.

As one of many CIM approaches, Traditional Chinese medicine (TCM) uses acupuncture to treat sleep disorders [27]. Music therapy, which is often used as an independent approach, is also used in the context of TCM. Although it is not included in the modern five pillars of TCM, it has historically played an important role in TCM therapy [28, 29]. In addition to references to music therapy in the classical texts, the close connection between music and medicine also emerges from the ancient Chinese characters. The ancient character for medicine is made up of the characters for music and herbs [30]. Different settings and musical instruments are used in music therapy, e.g. the use of a monochord [31]. The discovery of the monochord is attributed to Pythagoras of Samos and was used to research physical and music-theoretical phenomena [32]. Today, monochords are used particularly in music therapy and to accompany meditation [33–35]. They have different properties and vary in size tuning. One specific type of monochord is the I Ging Monochord®, with a concert pitch A tuned to 432 Hz and a resulting c of 128 Hz and C of 64 H. Furthermore, it allows concepts from TCM and the Yi Jing to be implemented acoustically [36, 37].

To date, there is a lack of high-quality randomized controlled trials (RCTs) investigating the efficacy of acupuncture in elderly patients. In the German S3 guideline “Non-restorative sleep/sleep disorders” of the German Society for Sleep Research and Sleep Medicine (DGSM), acupuncture and music therapy are listed as possible treatment options among the non-drug therapy methods [21]. According to the currently available evidence, various systematic reviews and meta-analyses show evidence for the efficacy of acupuncture in sleep disorders [38–40]. However, due to the poor quality of the original studies, the S3 guideline does not provide a conclusive recommendation. Several meta-analyses and systematic reviews on the use of music therapy for sleep disorders in adults conclude that music therapy possibly has a positive effect. However, the reviews also point out that the study quality of the original studies is poor [41–43]. Additionally, as geriatric patients still are often excluded from these studies, the results are therefore not necessarily transferable [44]. Therefore, there is a lack of high-quality randomized controlled trials (RCTs) investigating the efficacy of acupuncture or music therapy in elderly patients with sleeping disorders.

This study aims to identify the effect of a multimodal therapy approach based on Traditional Chinese Medicine on sleep quality in the elderly. Therefore, both the single components acupuncture and music therapy as well as the multimodal combination will be examined by using a factorial study design within a randomized controlled trial, embedded in the setting of an outpatient clinic. Therefore, the study is based on the following research questions:

1. *Quality of sleep (primary outcome):* How effective is each single intervention and the multimodal therapy approach in improving sleep quality in the elderly?
2. *Health-related quality of life and depression (secondary outcomes):* How does every single intervention and the multimodal therapy approach affect the participants’ health-related quality of life and depression?
3. *Neurovegetative Activity (secondary outcomes):* How is the heart rate variability affected by the interventions?
4. *Interviews and bodily sensations (secondary outcomes):* How do patients and providers perceive the single intervention and the multimodal therapy approach?
5. *Medication (secondary outcome):* How does every single intervention and the multimodal therapy approach influence (sleep) medication of patients?
6. *Safety/adverse effects:* How safe is every single intervention and the multimodal therapy approach? Do they lead to any adverse events?

## Methods and design

This protocol for a randomized controlled trial is reported in accordance with the SPIRIT statement [45, 46].

### Study setting

The trial is mono-centered and is conducted at our outpatient clinic for acupuncture and Chinese Medicine at the Institute of General Practice and Interprofessional Care, University Hospital Tübingen, Germany.

### Inclusion criteria for participants

Patients eligible for inclusion into the trial must be aged either 70 years or older with geriatric typical multimorbidity, or aged over 80 years. Furthermore, they need to suffer from sleeping disorders, measured by a Pittsburgh Sleep Quality Index (PSQI) global score of five or more [47]. Additionally, their German language skills and hearing ability have to be sufficient (simple ear examination).

### Exclusion criteria for participants

Patients are excluded if their health does not allow to take part, e.g. with cognitive impairment or dementia (MoCA Score ≤ 18), hemorrhages that required treatment within the last 6 months or coagulopathy of clinical relevance like hemophilia, acute mental disorders (e.g. imminent suicidal tendency or psychosis), severe disease (e.g. end of life care in case of cancer), acute or severe dermatosis (e.g. infectious erysipelas), contact allergies (e.g. nickel, chrome, silicone), needle phobia, no consent of participation.

### Eligibility criteria for therapists

The acupuncture intervention was developed based on internal evidence (clinical expertise of the study team) building on previous studies [48, 49], traditional teachings and acupuncture theory. The acupuncture sessions are provided by trained acupuncturists: medical doctors with specialization in acupuncture or by a medical doctoral student with comparable training and qualification (at least ≥ 200 h of training) under supervision. Music therapy is performed by qualified medical personnel with training in monochord playing. All therapists must be trained in both interventions to be able to apply the multimodal intervention.

### Interventions

We conduct a confirmatory non-blinded randomized controlled trial with a two-factorial design. This leads to four groups. Table 1 shows the factorial (two between-patient factors) design.

**Table 1:** Study design of ELAMUS

2 × 2-design	Music therapy	No Music Therapy
Acupuncture	Multimodal therapy approach (AM)	Acupuncture (AC)
No Acupuncture	Music therapy (MU)	Treatment as usual plus evidence-based information (TAUi)

The interventions are reported according to the STRICTA guidelines [50]. All participants receive printed, evidence-based materials on sleep disorders during their initial visit to the study site. the materials are specifically designed for elderly people, e.g. in a bigger font.

#### Intervention 1 (Treatment as Usual plus evidence-based information, TAUi

For intervention 1, participants will not get further treatment during the study period. However, they will receive five complimentary treatment sessions after completion of their study participation.

#### Intervention 2 (Acupuncture, AC)

In addition to TAUi, participants of this group will receive eight acupuncture treatments over 12 weeks. A single session will take around 30 min during which the patient is lying supine on a treatment bed. The acupuncture intervention follows a semi-standardized point selection. It encompasses three fixed acupoints (Shaohai H3 少海, Anmian HN 54 安眠, Shenting GV 24 神庭) and up to four points, which are individually chosen upon the guiding symptoms of the patients. In total, a maximum of 12 needles are used, chosen by the therapist as an individualized tailored TCM intervention. For all acupuncture sessions, sterile, silicone coated, single-use filiform acupuncture needles, with a length of 25 mm and a diameter of 0.25 mm each, are used. The manufacturer brand was not defined. Angle and depth of insertion is point specific according to acupuncture theory [51]. A de qi response is sought for the majority of the points but is not necessary, no further stimulation is applied during the needle retention time of 20 min. No other stimulation methods, such as moxibustion or electroacupuncture, are used. The well-being of the patient over the session time is observed by the therapist.

#### Intervention 3 (Music therapy, MU)

For intervention 3, participants will receive eight individual live receptive music therapy sessions over 12 weeks, in addition to TAUi. Each session lasts around 30 min with the patient lying on a treatment couch. For the music therapy, the patients receive a 15–20 min exposure to a I Ging monochord®, manufactured by Musikwelt Anklang, Germany [37]. The monochord is placed in an oblique row at the feet side of the treatment couch, approximately in a 1.5 m distance to the patient. The well-being of the patient over the session time is observed by the therapist, in case of agitation the monochord play can be adjusted to just one side of the instrument (personalization).

#### Intervention 4 (Multimodal therapy approach, AM)

Intervention 4 combines intervention 1, 2 and 3 (TAUi, AC and MU). Participants therefore receive both treatments simultaneously as described above, within 8 session over 12 weeks, each session lasting around 30 min. After placing the needles (see intervention 2), the therapist then starts playing the monochord (see Intervention 3).

For all intervention groups, reminders via telephone or e-mail are used in case a participant tends to miss their pre-scheduled appointments. Due to the health care services setting, there are no restrictions or prohibitions in concomitant care. However, participants are asked to report changes in routines or medications. Patients may withdraw from the study at any time without giving a reason.

### Outcomes

The outcomes will be measured three times, during a baseline measurement (T0), after completion of the intervention period of 12 weeks (T1, primary confirmatory analysis), and after the 12 weeks follow-up (T2).

The primary outcome of this study is improvement in sleep quality assessed via the Pittsburgh Sleep Quality Index (PSQI) global score [47, 52, 53] at T1. The PSQI is widely used in research and clinical practice with geriatric patients and consists of 19 items across seven components that form a global score [47, 54]. In addition, the following parameters will be collected as secondary outcomes via the following measurement tools: Depression (Geriatric Depression Scale, GDS-15) [55], Health-Related Quality of Life (SF-12) [56], Neurovegetative activity (Heart Rate Variability Measurement, HRV). Medication is assessed by medication plan or patient self-report. In addition, an accompanying process evaluation is carried out. A qualitative analysis of approximately *n* = 9–12 interviews with patients of the acupuncture and music therapy groups (*n* = 3–4 each for AC, MU, AM) and *n* = 2 interviews with the providers will be conducted [57]. The aim of the qualitative interviews is to gain insights into the individual experiences of the patients and sleep quality. Additionally, bodily sensations are assessed by drawings on a body outline for *n* = 20–25 patient cases (*n* = 7–8 each for AC, MU, AM) and the Phenomenology of Consciousness Inventory (PCI) [58]. The sample of patients for the process evaluation are chosen according to a purposeful sampling approach by the providers [59]. Tables 2 and 3 show a summary of the study’s outcomes.

**Table 2:** Outcome measurements (quantitative)

Primary outcome	Measurements
Sleep quality	Pittsburgh Sleep Quality Index (PSQI) (global score)
**Secondary outcomes**	
Depression	Geriatric Depression Scale (GDS-15)
Health-related Quality of Life	Short-Form-Health-Survey (SF-12)
Medication	Medication plan or patient self-report
Neurovegetative activity	Heart Rate Variability (HRV)

**Table 3:** Outcome measurements (process evaluation)

Secondary outcomes	Measurements
Qualitative evaluation	Qualitative interviews with patients (AC, MU, AM)
	Qualitative interviews with providers
Bodily sensations	Drawing of bodily sensations (AC, MU, AM)
	Phenomenology of Consciousness Inventory (PCI) (AC, MU, AM)

**Fig. 1:**
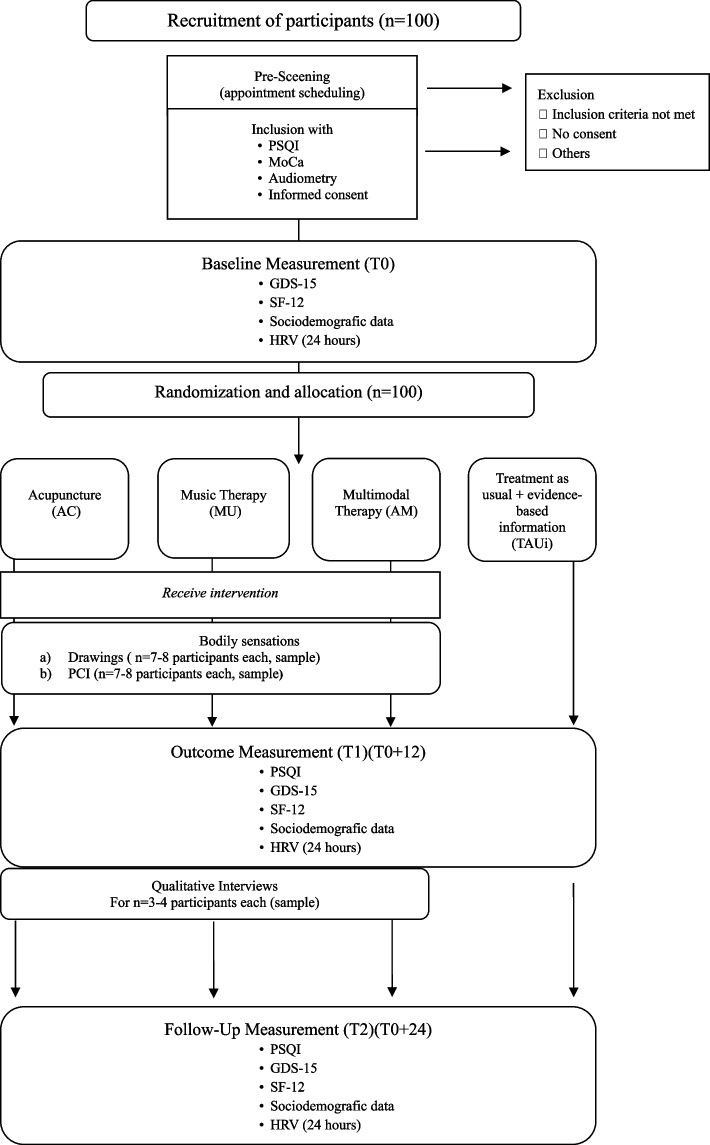
Participant timeline. PSQI: Pittsburgh Sleep Quality Index, MoCa: Montreal Cognitive Assessment, GDS-15: Geriatric Depression Scale, SF-12: Short-Form-Health-Survey, HRV: Heart Rate Variability

Figure 1 shows the participants’ timeline over the course of the study.

### Sample size

The study is designed to detect a minimal clinically important difference of 3 points on the PSQI global score [47] between groups with sufficient power. With an estimated standard deviation of 3.5 points on the PSQI global score, a minimal power of 80% and a dropout rate of up to 20%, mixed analysis of variance (mixed ANOVA) requires an estimated number of *n* = 100 participants, i.e. *n* = 25 participants per group.

### Recruitment

Participants are recruited via different channels: a press release was sent to local newspapers and radio channels. Additionally, printed flyers were handed out at local meeting points, events and courses for the elderly. Furthermore, the related local family practices of our Institute were asked to present the flyer to eligible patients and to display it in their practice.

### Allocation

The participants are allocated to the groups by using block randomization with randomly varying block lengths of 4, 8 or 12. The randomization list is set out by HC who is not involved in patient recruitment or data collection by using R software. The list is password protected, and only the biometrician has access to it. After patients are enrolled by KR, written consent has been received and the initial data collection has been completed, participants are centrally randomized by HC. The allocation is received via e-mail and communicated to the patients face-to-face (groups AC, MU, AM) or telephone (group TAUi).

#### Data collection, management, and analysis

Data for the patient-reported outcomes will be collected pseudonymized via paper and pencil questionnaires, filled out by the patients, at the three measurement time points. Adverse events will be documented at each measurement and/or intervention session. The neurovegetative activity measurements will be started after the questionnaires have been filled out: the therapist starts the recording after placing the heart rate variability recorder (CardioScout Multi ECG System, SR-Medizinelektronik, Germany) at the patients’ chest. The patients themselves take off the device at home after 24 h and send the device back (pre-paid envelope). Additionally, they are asked to fill out an activity protocol (e.g. sports, medication, etc.) during these 24 h at home and send it back with the device. The paper-based data will be stored for ten years in a secured steel locker only accessible by the study administrator.

All paper-based data will be typed into the study’s server-based database system (REDCap) by the study team and all study data are stored for ten years, using REDCap electronic data capture tools hosted at the University Hospital Tübingen [60, 61].

Via a secured VPN server, the pseudonymized data stored on the HRV devices are sent to JVG, who processes the data, e.g. data preparation for data analyses. These are then sent back via the secured VPN sever and statistically processed, analyzed and interpreted by JW, JVL, CK and KR. A Data Monitoring Committee is not set up and not required.

All outcome measures will be analyzed on the basis of the intention-to-treat principle. This means that all randomized participants are included in the analysis in the groups to which they were originally assigned, regardless of whether a complete data set is available or whether the study is conducted according to the protocol. Missing values will be multiply imputed.

Data of primary outcome PSQI and secondary outcomes GDS-15, SF-12 will be analyzed via analysis of variance (mixed between-within ANOVA). After this Omnibus testing, post-hoc t-tests for paired samples will be conducted, if the ANOVA reveals significant results. Bonferroni correction will be applied to correct for multiple testing. All variables (sociodemographic data, PSQI, GDS-15, SF-12) will be visualized and proportions will be descriptively analyzed. HRV data analysis will employ an exploratory approach due to the unknown effects of acupuncture and music therapy on the HRV of geriatric participants. This analysis will cover both frequency domain (e.g., HF and LF power) and time domain measures (e.g., RMSSD, SDNN, pNN50), which have shown to be important parameter with regard to sleep and HRV [62]. We will perform both within- and between-subject comparisons across the three measurement points and among the four intervention groups. Additionally, sensitivity analyses will be carried out to assess the robustness of our findings. Qualitative data will be analyzed exploratory using qualitative content analysis according to Kuckartz [63]. Medication and bodily sensations will be analyzed exploratory.

## Ethics and dissemination

The study got approval by the local Ethics committee (Ethics committee of the University and Faculty of Medicine Tübingen, 144/2023BO). Any important protocol changes will be adequately communicated via e-mail to the sponsor, the Ethics committee, any participant and respectively adapted within the trial registry.

Informed consent will be obtained from any potential participant to the study team (see Appendix). Personal data will be pseudonymized during the first contact call and during any of the sessions and measurement appointments. Data will be stored pseudonymized in a secured steel locker within the study venue and digitally stored pseudonymized on the University Hospital’s cloud server, only accessible for the direct study personnel who will be conducting the data analyses.

There is an active insurance covering travels to the study venue as well as potential harms through the intervention for every potential participant.

Author eligibility guidelines will be strictly followed for dissemination in presentations on scientific congresses and scientific publications. The results will be communicated by the sponsor and funding institution to the broad public in form of press releases, booklets or events.

## Discussion

This study’s aim is to analyze the effectiveness of a multimodal therapy intervention, combing acupuncture and music therapy, as well as the effectiveness of both the interventions separately on sleeping disorders in the elderly. The 2 × 2 factorial design strongly enables to observe the effect of any intervention and combination in comparison.

Systematic reviews and meta-analyses indicate that acupuncture and music therapy (each as individual procedures) generally have a positive effect on the treatment of sleep disorders [38–43]. The interventions are expected to have no severe side effects and to be effective and safe non-pharmacological approaches to support sleep quality in elderly people, based on TCM principles. Lack of blinding is a limitation of the study. This is due to the nature of the interventions, as music therapy cannot be blinded in our setting and no sham acupuncture is used. Recent publications presented evidence that shows that none of the sham acupuncture procedures (neither penetrating nor non-penetrating needles) are inert [64]. Insertion of an acupuncture needle anywhere on the body can stimulate local blood flow, increase local immune responses and induce analgesic responses [65–67]. Consequently, the US National Center for Complementary and Integrative Health “strongly discourages researchers from submitting research comparing clinical outcomes of verum and sham acupuncture” [68]. Furthermore, this is a clinical study in health care services research setting and focusing on outpatients. Consequently, context factors to reach a high internal validity (e.g. like medication or other therapies of study participants) are not tracked like in a stationary study and only listed to the three points of measurement and around the Heart Rate Variability measurement. Instead, results are expected to show a high external validity.

This study can provide valuable insights into the effectiveness of non-pharmacological interventions for sleep disorders among the elderly, contributing to a more personalized and holistic approach in geriatric healthcare.

### Supplementary Information

Supplementary Material 1: Supplementary Table S1: Study overview according to the WHO Trial Registration Data Set

## Data Availability

The datasets generated and/or analyzed during the current study will not be publicly available due to data protection regulations but are available from the corresponding author on reasonable request in accordance with the institutional regulations and the General Data Protection Regulation.

## Abbreviations

AC: Acupuncture intervention group
AM: Multimodal intervention group (combination of acupuncture and music therapy)
ANOVA: Analysis of variance
ARCIM: Academic Research in Complementary and Integrative Medicine
CIM: Complementary and Integrative Medicine
GDS-15: Geriatric Depression Scale
HRV: Heart Rate Variability
MU: Music therapy intervention group
PCI: Phenomenology of Consciousness Inventory
PSQI: Pittsburgh Sleep Quality Index
SF12: Short-Form-Health-Survey, 12 item version
TAUi: Treatment as usual plus evidence-based information intervention group
TCM: Traditional Chinese Medicine
