# Supplementary material for: A randomized controlled trial of acupuncture and receptive music therapy for sleep disorders in the elderly—ELAMUS: study protocol
Source: BMC Complement Med Ther. 2024 Aug 2;24:295. doi: 10.1186/s12906-024-04581-4 (PMC11295593; doi:10.1186/s12906-024-04581-4)
Supplement: Supplementary file 1 — Supplementary Material 1: Supplementary Table S1: Study overview according to the WHO Trial Registration Data Set [file 12906_2024_4581_MOESM1_ESM.docx]

**Supplementary Material**

## Supplementary Table 1: Study overview according to the WHO Trial Registration Data Set

| **Data category** | **Information** |
| --- | --- |
| Primary registry and trial identifying number | German Clinical Trials Registry, DRKS00031886 |
| Date of registration in primary registry | 17 May, 2023 |
| Secondary identifying numbers | - |
| Source(s) of monetary or material support | Karl and Veronica Carstens Foundation |
| Primary sponsor | University Hospital and Faculty of Medicine Tübingen, Geissweg 3, 72076 Tübingen, Germany |
| Secondary sponsor(s) | - |
| Contact for public queries | CK, carina.klocke@med.uni-tuebingen.de |
| Contact for scientific queries | CK, Institute of General Practice and Interprofessional Care, University Hospital and Faculty of Medicine Tübingen, Tübingen, Germany |
| Public title | A randomized controlled trial of acupuncture and receptive music therapy for sleep disorders in the elderly (ELAMUS) |
| Scientific title | A randomized controlled trial of acupuncture and receptive music therapy for sleep disorders in the elderly (ELAMUS) |
| Countries of recruitment | Germany |
| Health condition(s) or problem(s) studied | Sleeping disorders and insomnia in the elderly |
| Intervention(s) | Interventions are applied within 8 sessions over 12 weeks:  *Intervention 1*: Acupuncture (AC) *Intervention 2*: Music therapy (MU)  *Intervention 3*: Multimodal combination of AC and MU (AM)  *Control*: Treatment as usual plus evidence-based information (TAUi) |
| Key inclusion and exclusion criteria | *Ages eligible for study*: ≥ 70 years and age-related multimorbidity or ≥ 80 years *Sexes eligible for study*: both *Accepts healthy volunteers*: no *Inclusion criteria*: sleeping disorder (PSQI global score ≥ 5), ability to come to the study venue, sufficient language skills *Exclusion criteria*: Cognitive impairment (MoCA Score ≤ 18), hemorrhages that required treatment within the last 6 months or coagulopathy of clinical relevance (e.g. hemophilia, acute mental disorders (e.g. imminent suicidal tendency or psychosis), severe disease (e.g. end of life care in case of cancer), acute or severe dermatosis (e.g. infectious erysipelas), contact allergies (e.g. nickel, chrome, silicone), needle phobia, no consent of participation |
| Study type | Interventional randomized controlled trial *Allocation*: randomized *Intervention model*: 2x2 design *Masking*: none *Primary purpose*: therapy |
| Date of first enrolment | May 2023 |
| Target sample size | 100 |
| Recruitment status | Recruiting |
| Primary outcome(s) | sleep quality, assessed via Pittsburgh Sleep Quality Index (PSQI) (global score) |
| Key secondary outcomes | Depression (Geriatric Depression Scale, GDS-15), Health-Related Quality of Life (SF-12), Neurovegetative Activity (Heart Rate Variability Measure, HRV), process evaluation (qualitative interviews with patients, bodily sensations) |
